# Sustainable management of riverine N_2_O emission baselines

**DOI:** 10.1093/nsr/nwae458

**Published:** 2024-12-11

**Authors:** Shuo Wang, Wei Zhi, Shengjie Li, Tao Lyu, Guodong Ji

**Affiliations:** Key Laboratory of Water and Sediment Sciences, Ministry of Education, Department of Environmental Engineering, Peking University, Beijing 100871, China; The National Key Laboratory of Water Disaster Prevention, Yangtze Institute for Conservation and Development, Key Laboratory of Hydrologic-Cycle and Hydrodynamic-System of Ministry of Water Resources, College of Hydrology and Water Resources, Hohai University, Nanjing 210024, China; Department of Biogeochemistry, Max Planck Institute for Marine Microbiology, Bremen 28359, Germany; School of Water, Energy and Environment, Cranfield University, Cranfield MK43 0AL, UK; Key Laboratory of Water and Sediment Sciences, Ministry of Education, Department of Environmental Engineering, Peking University, Beijing 100871, China

**Keywords:** nitrous oxide, emission factor, hotspots, sustainable, greenhouse gases

## Abstract

The riverine N_2_O fluxes are assumed to linearly increase with nitrate loading. However, this linear relationship with a uniform EF_5r_ is poorly constrained, which impedes the N_2_O estimation and mitigation. Our meta-analysis discovered a universal N_2_O emission baseline (EF_5r_ = k/[NO_3_^−^], k = 0.02) for natural rivers. Anthropogenic impacts caused an overall increase in baselines and the emergence of hotspots, which constitute two typical patterns of anthropogenic sources. The k values of agricultural and urban rivers increased to 0.09 and 0.05, respectively, with 11% and 14% of points becoming N_2_O hotspots. Priority control of organic and NH_4_^+^ pollution could eliminate hotspots and reduce emissions by 51.6% and 63.7%, respectively. Further restoration of baseline emissions on nitrate removal is a long-term challenge considering population growth and declining unit benefits (ΔN-N_2_O/N-NO_3_^−^). The discovery of EF lines emphasized the importance of targeting hotspots and managing baseline emissions sustainably to balance social and environmental benefits.

## Teaser

The priority control of organic and NH4+ pollution could rapidly eliminate global riverine N2O hotspots and reduce emissions by half. The further restoration of baseline emissions on nitrate removal is a long-term challenge.

## INTRODUCTION

In the Anthropocene epoch, agriculture and rapid urbanization completely alter the natural nutrient conservation mechanism under the impact of clearing natural vegetation and introducing excessive nutrients. Increasing N availability in global rivers leads to unintended environmental consequences, including increased nitrous oxide (N_2_O) emissions [1,2]. Global rivers thereby become a significant anthropogenic source for N_2_O emissions. Estimates indicate that global riverine N_2_O emissions have experienced a 4-fold increase since the 1900s [2]; this rate of increase is three times faster than terrestrial ones [2], contributing 10%–30% to the overall anthropogenic N_2_O budget [3,4].

In the estimation and management of indirect N_2_O emissions (The Intergovernmental Panel on Climate Change (IPCC)), riverine N_2_O fluxes are commonly assumed to linearly increase with nitrate loading on the basis of the emission factor EF_5r_ ([N_2_O]/[NO_3_^−^]) [5,6]. EF_5r_ has been constantly refined by the IPCC from 0.75% (1998) to 0.25% (2006) and to 0.26% (2019). However, recent studies have continued to argue that the current default EF_5r_ may either overestimate or underestimate the riverine N_2_O emissions [6–8]. Although riverine N_2_O emissions tend to increase with N inputs, this linear relationship with a uniform EF_5r_ (0.26%) is often weak or non-existent [9,10]. Moreover, the observed EF_5r_ is poorly constrained due to the high heterogeneity of global rivers, ranging from 0.005% to 7% [4,11]. As a result, uncertainties will inevitably widen when extrapolating the simplistic factor for the global estimation. Additionally, our understanding of riverine N_2_O emissions remains fragmented with a bias towards N-rich rivers, yet no clear large-scale pattern has been identified for global rivers [9]. This simplistic and fragmented understanding thereby hinders our ability to comprehend the underlying mechanism of riverine N_2_O emissions and quantify human impacts. This, in turn, impedes the development of effective mitigation strategies.

Agricultural production, animal husbandry, industrial and domestic sewage are the major sources of general nitrogen and organic pollution in rivers. The continuous urbanization, increasing food demand and widespread deoxygenation in warming rivers will further increase N_2_O emissions [12–15]. The current emission factor EF_5r_ ([N_2_O]/[NO_3_^−^]) emphasizes the importance of reducing nitrate in N_2_O emission mitigation. However, the broad range of EF_5r_ underscores the complexity of microbial processes and environmental factors regulating riverine N_2_O emissions. Riverine production of N_2_O greatly depends on microbial activity, particularly nitrification, nitrifier denitrification (NDN) and denitrification processes [16]. Incomplete denitrification is commonly assumed to be the dominant pathway regulating N_2_O production and consumption in rivers [6,17]. However, denitrification activity in aquatic ecosystems is generally limited by the supply of electron donors (organic matter) rather than NO_3_^−^ [18]. Whether the introduction of excess nitrate can linearly increase N_2_O production, as well as the role of organic carbon, requires further investigation. Additionally, recent research has emphasized nitrification as a non-negligible source of N_2_O emissions in rivers and lakes [19,20]. NH_4_^+^-derived pathways were even proven to be the dominant N_2_O sources in low-order agricultural streams around the world [21]. These findings collectively challenge the prevailing management priorities regarding nitrate. Therefore, we hypothesize that organic pollutants and ammonia may also play an important role in the management of riverine N_2_O emissions, which have been underestimated in previous models.

It is worth noting that the IPCC method provides policymakers with convenient and easy-to-use parameters for regional and global N_2_O emission estimations [22]. Consequently, it has been widely applied with a large-scale dataset [23–46]. Previous studies have proposed a variety of non-linear models, including efficiency loss models and exponential models, for refinement of emission factor estimates [7]. The extensive range of EF_5r_ presents an opportunity for the extraction of valuable insights through data mining. To do so, we conducted a comprehensive global meta-analysis including 3047 *in situ* measurements across different river types (natural, agricultural and urban rivers) to explore the intrinsic nature of EF_5r_. Seasonal measurements of N_2_O fluxes and isotopic signatures were also performed in rivers impacted by anthropogenic activities (agriculture and urbanization) to determine the prevailing microbial processes and predominant environmental drivers. Finally, in this study, a global distribution map of N_2_O hotspots with effective management strategies was generated. This study can enhance our understanding of how human activities impact global riverine N_2_O emissions and address the questions of how much of these emissions can be mitigated and how to do so most effectively.

## RESULTS

### Universal EF lines for global rivers

**Figure 1.**
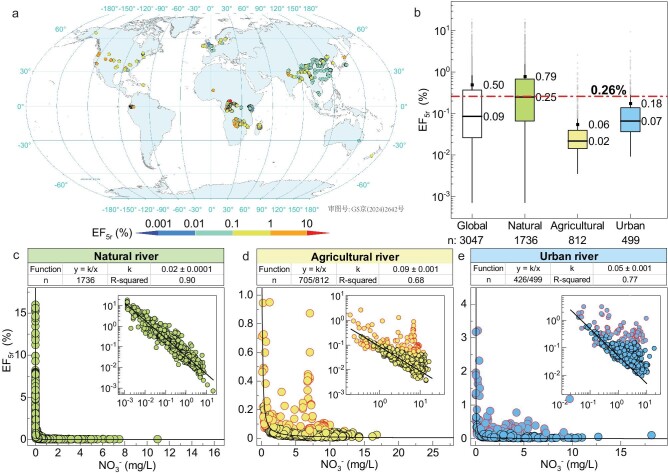
Location and characteristics of EF_5r_ used in the meta-analysis. (a) Geographical distribution of EF_5r_ from the global riverine sample sites. (b) EF_5r_ of global rivers and variations across different river types (natural, agricultural and urban rivers). The boxes are bounded by the 25th and 75th percentiles. The whiskers represent 1.5 × the interquartile range, and the solid line denotes the median. The black dots denote arithmetic means, and the gray dots denote outliers. (c-e) EF lines with k values indicating nutrient levels and basic N_2_O emissions in the different rivers. The model function y = k/x was employed for fitting. In each iteration, a residual analysis was performed to exclude any points deviating from the baseline (3σ rule) until there were no outliers in the fitted curve. n indicates the points without outliers/total sampling points.

The determination of N_2_O emissions for highly heterogeneous rivers has long remained a contentious issue. The IPCC adopted a value of 0.26% (n = 91) as a representation of the global riverine emission factor [47]. Through the analysis of a comprehensive dataset comprising 3047 observations, it was revealed that EF_5r_ varies by five orders of magnitude (Fig. 1a), with an average value of 0.50% (Fig. 1b), which is greater than that adopted by the IPCC. Importantly, our results indicated that the EF_5r_ value adopted by the IPCC cannot be universally applied across all rivers, because EF_5r_ exhibits significant variations across different river types. The EF_5r_ of natural rivers without human influence (0.79%) was notably greater than that of agricultural rivers (0.06%) and urban rivers (0.18%), leading to both overestimation and underestimation of EF_5r_ in specific river categories. These findings emphasize the importance of considering the distinct characteristics of different river types when estimating N_2_O emissions.

Furthermore, it was revealed that EF_5r_ decreased with increasing NO_3_^−^ concentration (Fig. 1c), precisely conforming to the inversely proportional function EF_5r_ = k/[NO_3_^−^] (R^2^ = 0.90) for natural rivers, hereafter referred to as the EF baseline. A k value of 0.02 represents situations without human disturbance. At the baseline, N_2_O concentrations are insensitive to further increases in nitrate levels, but are determined by the k value (EF_5r_ = k/[NO_3_^−^] = [N_2_O]/[NO_3_^−^], that is [N_2_O] = k). Iterative analyses were further conducted on individual watersheds (Fig. S1a–j), and the k values were positively correlated with the average nitrate concentration in rivers (Fig. S1k, R^2^ = 0.78, *P* < 0.001). Therefore, the k value could serve as a reference for the overall nutrient level and basic N_2_O emissions in rivers. In other words, the average nitrate concentration determines the basic N_2_O emissions in rivers.

**Figure 2.**
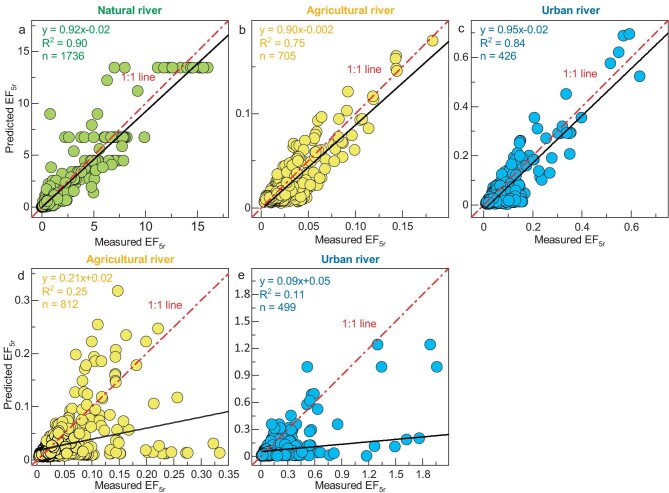
Measured versus predicted EF_5r_ values. (a-c) The riverine sampling points without outliers. (d, e) All the sampling points. The red dotted line denotes the 1:1 line. The black line denotes the linear regression fit, and R^2^ is the coefficient of determination.

Regarding agricultural and urban rivers, anthropogenic impacts caused an overall increase in their baselines and the emergence of hotspots (Fig. 1d and e, Figs S2–S3). To obtain baselines, outliers were excluded according to the 3σ rule in each fitting iteration (Tables S1–S2). The k values of agricultural and urban rivers increased to 0.09 and 0.05, respectively, which is consistent with the increase in nutrient levels. The plot of measured EF_5r_ versus predicted EF_5r_ values illustrates the reliability of EF lines (Fig. 2a–c). Notably, most outliers that deviated from the baseline became N_2_O hotspots in agricultural (11%) and urban rivers (14%). Several outliers occurred below the fitting line, especially in agricultural rivers, which are probably caused by natural factors (e.g. waterfalls or rainstorms) or human activities (e.g. irrigation). When anthropogenic impacts exist, EF_5r_ significantly deviates from the baselines and cannot be accurately predicted with nitrate as the only variable (Fig. 2d and e). Overall, the baseline emissions and hotspots constitute two typical patterns of anthropogenic sources in global rivers. To reveal the underlying mechanism of the two typical N_2_O emission patterns, regional investigations based on isotopes and meta-analyses of the physicochemical parameters of global rivers were performed.

### N_2_O baseline emissions

During the initial phase of the baselines, even minor amounts of NO_3_^−^ were accompanied by a certain N_2_O_equilibrium_ level, resulting in extremely high emission factors near the zero point, especially for natural rivers (Fig. 1c). Subsequently, EF_5r_ decreased with increasing NO_3_^−^ concentration. Therefore, an increase in nitrate does not necessarily lead to increased N_2_O emissions. Similar phenomena have been observed in rivers and estuaries and have been attributed to biological saturation [7,13]. To reveal the underlying mechanism, the microbial sources of N_2_O production in anthropogenically impacted rivers were quantified by isotopic approaches (Figs S4–S6), which can provide an insight into N_2_O production and reduction mechanisms [48–50]. In Haihe River basin, the EF baseline was established with the function y = 0.045/x (Fig. S6a, R^2^ = 0.91). Isotopic signatures of N_2_O, δ^15^N^Bulk^, δ^18^O and δ^15^N^SP^ were combined to separate N_2_O microbial sources in 3D isotope space (Fig. S6b). For the sampling points around the baseline, nitrification significantly contributed to N_2_O emissions (51.7%), thereby a decrease in the NH_4_^+^/NO_3_^−^ ratio led to a decrease in EF_5r_ ([N_2_O]/[NO_3_^−^]) (Fig. S6c and d). Similarly, denitrification accounted for an average contribution of 39.2% to N_2_O emissions for baseline points (Fig. S6c). As NO_3_^−^ reduction typically occurs only when there are sufficient electron donors [18,51], excessive nitrate is rendered inert nitrogen. Therefore, the decreasing dissolved organic carbon (DOC)/NO_3_^−^ ratio with nitrate limits the production of N_2_O, further resulting in a decrease in EF_5r_ (Fig. S6e). From the regional to global scales, the NH_4_^+^/NO_3_^−^ and DOC/NO_3_^−^ ratios jointly determine the EF_5r_ according to significant linear relationships, and the inversely proportional decreases in the NH_4_^+^/NO_3_^−^ and DOC/NO_3_^−^ ratios with increasing NO_3_^−^ finally determine the distribution of the EF baselines (Fig. S7).

**Figure 3.**
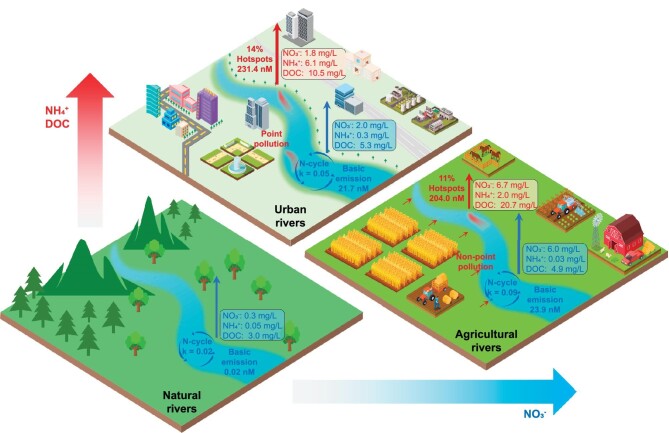
Natural and anthropogenic N_2_O emissions. Anthropogenic sources constitute the primary component of N_2_O emissions in global rivers and are mainly manifested as two patterns: baseline emissions and localized hotspots. The k values of the EF lines for natural, agricultural and urban rivers were determined at 0.02, 0.09 and 0.05, respectively, indicating increasing nutrient levels and basic N_2_O emissions in the different types of rivers. The corresponding median ΔN_2_O concentrations were 0.02, 23.9 and 21.7 nM, respectively. The median NH_4_^+^, NO_3_^−^ and DOC concentrations for the basic emissions (blue) and localized hotspots (red) are shown for the different rivers. Additionally, the median ΔN_2_O concentration at localized hotspots reached 204.0 nM (11%) and 231.4 nM (14%) in agricultural and urban rivers, respectively.

**Figure 4.**
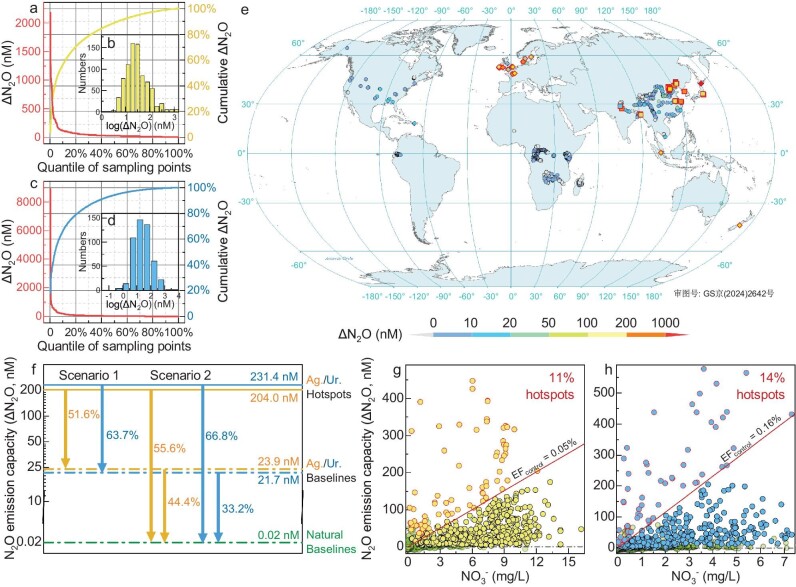
Effective management strategies. (a, c) ΔN-N_2_O concentration (red lines), and cumulative ΔN-N_2_O (yellow/blue lines) for different quantiles of sampling points; (b, d) Log-normal distribution of ΔN-N_2_O in agricultural and urban rivers; (e) Geographical distribution of N_2_O emissions (ΔN_2_O) in global rivers. The diamonds and squares with red borders denote the hotspots in agricultural and urban rivers, respectively. (f) Scenarios for the management of riverine N_2_O emissions. (g, h) Nitrate-based ΔN_2_O reduction model. The green, yellow and blue points denote the sampling points in natural, agricultural and urban rivers, respectively. The points with red borders indicate N_2_O hotspots, and the slope (ΔN-N_2_O/N-NO_3_^−^) represents the unit benefit of N_2_O emission reduction caused by treating a unit of nitrate.

The baseline N_2_O emissions were characterized by specific k values in the different types of rivers (Fig. 3 and Fig. S8). In natural rivers, the surrounding terrestrial vegetation and internal carbon/nitrogen cycle are the major sources of nitrogen (NH_4_^+^ and NO_3_^−^) and organic matter [52]. They are generally subject to the overall nutrient level in the system, with the total nitrogen (TN; comprising mainly nitrate) as one of the main limiting factors. Therefore, natural rivers effectively sequester and recycle N at relatively low nutrient levels (k = 0.02) due to the extremely limited N availability (median NO_3_^−^ = 0.3 mg/L). The difference in the N_2_O partial pressure (ΔN_2_O = N_2_O_water_–N_2_O_equilibrium_) was used to indicate the N_2_O emission capacity of global rivers. The natural source of riverine N_2_O emissions was negligible, with a median ΔN_2_O concentration of 0.02 nM. Geographically, most high-order natural rivers, such as the Napo River, Yellow River, Yangtze River and Ganges River, generally exhibit low levels of baseline N_2_O emissions, even functioning as sinks of atmospheric N_2_O (Fig. 4e). By contrast, agriculture and urbanization introduce excessive nutrients into rivers. The median NO_3_^−^ concentration for the agricultural baseline points was 6.0 mg/L, which is significantly greater than that of the other rivers (Fig. 3). Although it is difficult for inert nitrates to be directly reduced in aerobic rivers with insufficient electron donors, elevated nutrient levels could determine the primary productivity of phytoplankton and surrounding terrestrial vegetation, thereby promoting basic N_2_O emissions with an increased k value of 0.09. Similarly, the moderate TN level (median NO_3_^−^ = 2.0 mg/L) in urban rivers caused an increase in the k value to 0.05. The corresponding median ΔN_2_O concentrations in agricultural and urban rivers were 23.9 and 21.7 nM, respectively, which are significantly greater than those in natural rivers. It is worth noting that the baseline N_2_O emissions in agricultural rivers are significantly higher than those in urban rivers, largely due to the substantial leaching of fertilizer from agricultural non-point sources.

### Localized hotspots

The hotspots are generally derived from sudden increases in organic and NH_4_^+^ loads. In the case of the identified hotspots (Fig. S6a, Spring-CB1 and Autumn-CB5), organic pollution activates nitrate, leading to a significant increase in denitrification activity (Fig. S6c). Because NH_4_^+^ pollution often occurs simultaneously with organic pollution (Fig. S9), the introduction of NH_4_^+^ also significantly contributes to N_2_O production through nitrification and nitrifier denitrification. Therefore, sampling points where the NH_4_^+^/NO_3_^−^ and DOC/NO_3_^−^ ratios deviated from the fitting lines potentially became hotspots of N_2_O emissions (Fig. S6d and e). Globally, the NH_4_^+^ and DOC concentrations in hotspots were significantly greater than those at the baseline points, especially for the NH_4_^+^/NO_3_^−^ and DOC/NO_3_^−^ ratios (Fig. S8b–e).

Although only a few of the sampling points became hotspots in agricultural (11%) and urban (14%) rivers, the median ΔN_2_O concentrations reached 204.0 and 231.4 nM, respectively (Fig. 3 and Fig. S8), almost 10 times the basic emission levels. The hotspot emissions in urban rivers are more severe than those in agricultural rivers due to the greater impact of point source pollution in urban areas. Statistically, a log-normal probability distribution for the ΔN-N_2_O concentration was observed in agricultural and urban rivers (Fig. 4a–d), indicating the disproportionate contributions of hotspots at the tail of these frequency distributions. Therefore, targeting pollution control towards the high ends of these distributions would probably result in greater environmental gains per unit of management effort. In contrast to natural rivers, low-order streams contributed disproportionate amounts to N_2_O emissions due to the massive amounts of terrestrially derived carbon/nitrogen inputs (Fig. 4e). Agricultural N_2_O hotspots were mainly distributed in Asia and Europe, which exhibit intensively cultivated croplands and dense populations. Moreover, urban hotspots were significant in megacities, such as Beijing, Shanghai, Tokyo and New Delhi, while no significant urban hotspots were observed in developed countries, such as the USA, Canada and in Europe. Because the point source pollution in urban areas is comparatively more manageable than non-point source pollution in agricultural rivers, developed countries have basically eliminated hotspot emissions in urban rivers, but still experience hotspot emissions caused by agricultural non-point source pollution. Additionally, due to the lack of monitoring sites, potential hotspots in certain developing countries are not shown on the map.

## DISCUSSION

Anthropogenic perturbations in nitrogen flow have exceeded the proposed planetary boundary [53], leading to concomitant threats to agricultural productivity, food security, global climate and economic prosperity [54]. We are trying to explain how N_2_O is emitted from global rivers and how to reduce it using the simplest mathematical curve. The discovery of EF lines helps us better understand how human activities affect global riverine N_2_O emissions and aids in the development of effective management strategies.

### Riverine N_2_O emission patterns

The discovery of EF lines reveals the two typical patterns of anthropogenic N_2_O sources in global rivers: an overall increase in baselines and the emergence of hotspots. At the baseline, accurate predictions of EF_5r_ are feasible for rivers with the function EF_5r_ = k/[NO_3_^−^] (Fig. 2a–c). The riverine N_2_O concentration was insensitive to further increases in nitrate levels but was determined by the k value. The k value is generally determined by the overall nutrient level in rivers, mainly by the nitrate concentration. Therefore, nitrate removal is the key point for mitigating baseline N_2_O emissions. However, the existence of hotspots caused the measured EF_5r_ to significantly deviate from the baseline trend, and EF_5r_ cannot be accurately predicted with nitrate as the only variable (Fig. 2d and e). As we hypothesized, organic pollution and NH_4_^+^ play an important role in the riverine N_2_O emissions. Organic matter rapidly activates nitrate, accompanied by simultaneous NH_4_^+^ input (Fig. S9), leading to a significant increase in N_2_O emissions. In contrast to the basic N_2_O emissions with time lags, localized hotspots can exhibit excessive N_2_O emissions within a short time, where anthropogenic pollution might exceed the environmental carrying capacity, accompanied by irreversible changes and serious degradation in ecosystems (e.g. algal blooms and black-odor water). Therefore, restoring hotspots might return their original ecological functions and provide additional ecological benefits.

### Two scenarios for mitigating riverine N_2_O emissions

Two scenarios (Fig. 4f) were established to answer the following questions: How much can riverine N_2_O emissions be mitigated? How can they be mitigated more effectively?

**Scenario 1:** Restore the agricultural/urban hotspots (204.0/231.4 nM) to their baselines (23.9/21.7 nM) by controlling organic and NH_4_^+^ pollution. Despite similar nitrate concentrations between hotspots and baseline points, the excessive presence of DOC and NH_4_^+^ results in significant N_2_O emissions (Fig. 3 and Fig. S8). Therefore, controlling organic and NH_4_^+^ pollution can restore agricultural/urban hotspots to their baseline levels without further nitrate removal. As a result, restoring hotspots in agricultural (11%) and urban (14%) rivers could reduce N_2_O emissions by 51.6% and 63.7%, respectively. Even in agricultural rivers dominated by non-point source pollution, critical source areas often contribute most to the pollution load in the entire watershed.

**Scenario 2:** Restore the agricultural/urban hotspots and their baselines to the natural baselines (0.02 nM). In addition to organic and NH_4_^+^ pollution control, further nitrate removal is needed to completely eliminate N_2_O emissions in rivers (Fig. 3 and Fig. S8). In this case, restoring hotspots to the natural baselines in agricultural and urban rivers could reduce N_2_O emissions by 55.6% and 66.8%, respectively. This is only slightly higher than in Scenario 1 (51.6% and 63.7%), but was achieved at a greater cost for additional nitrate removal. Additionally, the further restoration of remaining baselines needs to manage more than 80% sampling points, but could only reduce N_2_O emissions by 44.4% and 33.2% in agricultural and urban rivers, respectively.

From the perspective of nitrate removal (Fig. 4g and h), the slope represents the benefit of N_2_O emission reduction achieved by treating a unit of nitrate (ΔN-N_2_O/N-NO_3_^−^). Obviously, the hotspots identified by the EF lines exclusively occurred in the upper left, the elimination of which provided the greatest unit benefit of 0.25% and 0.69% in agricultural and urban rivers, respectively (Table S4). Instead of mechanical restoration from the highest ΔN-N_2_O concentration, our model considered a more efficient mitigation strategy on the order of unit benefits (slope, ΔN-N_2_O/N-NO_3_^−^). Moreover, we found that the thresholds for N_2_O hotspot control (EF_Control_, ΔN-N_2_O/N-NO_3_^−^) in agricultural and urban rivers were 0.05% and 0.16%, respectively. These values can easily be applied as a reference for the identification and management of N_2_O hotspots. However, further restoration of the baseline points with decreasing slopes resulted in a steep decrease in the unit benefits (Table S4). Thereby, restoring the baseline points could only reduce N_2_O emissions by 44.4% and 33.2% in agricultural and urban rivers, respectively (Fig. 4f). In contrast to hotspots with severe organic pollution, nitrate removal at the baseline points also requires additional electron donors. Therefore, mitigation strategies for both agricultural and urban rivers should first focus on eliminating organic and NH_4_^+^ pollution at hotspots, while nitrate removal provided lower unit benefits for N_2_O mitigation but came at a greater cost.

### Sustainable management strategies

The sustainable management of N_2_O should integrate environmental, social and economic considerations into the decision-making process. According to the definition of EF_5r_ (IPCC), N_2_O emissions were assumed to linearly increase with nitrogen loading. In this case, nitrate removal is crucial for controlling N_2_O emissions. However, our results suggested that targeting hotspot elimination would probably result in greater environmental value. It is thereby recommended to first restore agricultural/urban hotspots to their baselines and then restore their baselines to the natural baselines. In other words, in regions with significant N_2_O hotspots, controlling organic and NH_4_^+^ pollution should be prioritized. Compared with nitrate, the treatment of organic and NH_4_^+^ pollution is easier and less costly. The priority control of organic and NH_4_^+^ pollution could rapidly eliminate N_2_O hotspots and halve its total emissions. This finding is of great significance to practical engineering and aids in the development of more effective management strategies.

The point source pollution in urban areas is comparatively more manageable. Based on data from the United Nations Sustainable Development Goals (SDG 6, clean water and sanitation) [55], the sewage collection rate is more than 90% in developed countries, thereby no significant urban hotspots were observed (Fig. 4e). By contrast, the collection rate is less than 60% in most developing countries, especially India (21%), which hosts a large population, thereby leading to the situation of being besieged by N_2_O hotspots (Fig. 4e). In the future, well-managed urbanization, including land intensification, the improvement of sewage collection and an expansion of wastewater treatment capabilities, will be favorable for rapidly eliminating hotspots in developing countries. However, managing non-point source pollution in rural areas is challenging. It has been reported that ∼60% of the fertilizer applied in soils leaches into stream systems [21]. Therefore, certain developed countries, such as Japan, New Zealand and those in Europe, still experience hotspot emissions caused by agricultural non-point source pollution. This phenomenon is more serious in developing countries. For example, China has long been the largest NH_4_^+^ fertilizer consumer in the world [21]; large numbers of agricultural hotspots have been observed in the Beijing-Tianjin region and the Yangtze River Delta. Currently, most measures are restricted and rarely implemented due to socioeconomic barriers, such as small farm size [56]. The continued urbanization leads to rural depopulation, both through rural-to-urban migration and natural aging, benefiting the ongoing increase of large-scale farming practices. It has been estimated that the proportion of large-scale cropland farms could increase from 9% in 2017 to 90% by 2050 in China, thus improving cropland nitrogen use efficiency from 43% to 54% [57]. Overall, agricultural centralization and the identification of critical source areas are important for managing N_2_O hotspots in agricultural watersheds [58].

The priority control of organic and NH_4_^+^ pollution could rapidly eliminate global riverine N_2_O hotspots and reduce emissions by one-half. After eliminating hotspots, the management of baseline emissions is notably constrained by social and economic development. Considering population growth and the massive demand for fertilizer, nitrate will inevitably be leached from intensive arable systems. For instance, the median NO_3_^−^ concentration at the agricultural baseline points was 6.0 mg/L, 20 times the natural level of 0.3 mg/L. Mitigating baseline emissions requires long-term investment but yields lower unit benefits. Fortunately, our EF lines demonstrate that the ecosystem can balance and withstand moderate anthropogenic pressure with increased k values. Moreover, too little N could lead to lower crop productivity and poor human nutrition. As a result, nitrate concentrations can remain at appropriate high levels within the environmental carrying capacity, where a greater N cycle and primary production favor rapid N removal and self-restoration.

## MATERIALS AND METHODS

### Data collection and sampling sites

A literature search was conducted using bibliographic databases (Web of Science, Google Scholar, etc.) for papers containing data on N_2_O concentrations and indirect N_2_O emission factors (EF_5r_) for global rivers (from 2000 to 2023) [23–46]. In total, our efforts identified ∼3000 river points with N_2_O, NO_3_^−^, NH_4_^+^ and DOC concentrations. In addition to literature data, N_2_O-related parameters from large river networks in China were also measured (https://thesis.lib.pku.edu.cn/). A list of the cited and measured data is provided in the Supplementary Data. To further reveal anthropogenic impacts on N_2_O emission, a seasonal measurement of N_2_O fluxes and isotope values in the Haihe River basin was conducted to reveal the underlying process and the main environmental regulators controlling EF_5r_. *In situ* measurements were conducted in two major rivers within the Haihe River basin, namely, the YongDing River and ChaoBai River, which are significantly impacted by urbanization and agriculture (Fig. S4). This region exhibits a continental monsoon climate characterized by an annual precipitation of ∼600 mm and an average annual temperature of 10−12°C [3]. Triplicate water samples were collected from rivers during ice-free periods, and seasonal sampling was performed during the spring (April), summer (July) and autumn (October) seasons of 2022. In total, 108 observations were obtained during the sampling campaigns. No abnormal hydrological variations or extreme weather events were recorded during the sampling periods.

### Establishment of the EF-line model

According to the river type, we divided the collected data into natural, agricultural and urban river data. Only field observations with original data were used for further fitting. The non-linear relationship between the EF_5r_ value and NO_3_^−^ concentration was fitted using the non-linear curve fit option in Origin 2020 via the Levenberg–Marquardt algorithm. The model function y = k/x was employed for fitting. A residual analysis was performed to exclude any points deviating from the baseline. In each iteration, sampling points with residual errors greater than three times the standard deviation were identified as outliers (3σ rule). Outliers were excluded, and the remaining points were again fitted at each time point until there were no outliers in the fitted curve (Tables S1–S2). EF lines of natural, agricultural and urban rivers were finally obtained via iterations (Figs S1–S3), with the outliers highlighted in red in the Supplementary Data. In addition, EF lines of individual rivers were obtained in the same way.

### Physicochemical parameters and dissolved N_2_O

A precalibrated portable multiparameter device (DZB-718, Leici, China) was used to record the water temperature, pH, dissolved oxygen (DO), oxidation-reduction potential and electrical conductivity (EC) simultaneously. Subsequently, all water samples were stored at 4°C during transport to the laboratory. Subsequently, the water samples were filtered through 0.22-μm polyethersulfone syringe filters. An ultraviolet spectrophotometer (UV5100, Shanghai, China) was employed for the determination of NH_4_^+^ (Nessler's reagent spectrophotometry method), NO_2_^−^ (diazamine coincidence spectrophotometry) and NO_3_^−^ (ultraviolet spectrophotometric method). The DOC concentration was determined using a total organic carbon (TOC) analyzer (TOC-L, Shimadzu, Japan). The dissolved concentrations of N_2_O in the freshwater samples were measured by an Agilent 7890B gas chromatograph (Agilent 7890B, μECD) using the headspace equilibrium method [59]. The temperatures of the oven, chromatographic column and the detector were kept constant at 70, 280 and 330°C, respectively. The dissolved N_2_O concentration in the water samples (N_2_O_water_) was calculated based on the partial pressure of N_2_O in the headspace and Henry's law constant for N_2_O (Eq. 1) [60]:


(1) \begin{eqnarray*}{{{\mathrm{N}}}_2}{{{\mathrm{O}}}_{{\mathrm{water}}}} = {{{\mathrm{C}}}_{\mathrm{g}}} \times \left( {\frac{{{{V}_g}}}{{{{V}_l}}}\ + {{{\mathrm{K}}}_{\mathrm{h}}}{\mathrm{RT}}} \right),
\end{eqnarray*}

where C_g_ is the measured N_2_O concentration in the headspace (nmol/L); N_2_O_water_ is the calculated dissolved N_2_O concentration (nmol/L); V_g_ and V_l_ are the gas and liquid volumes, respectively (mL); K_h_ is Henry's law constant, which is calculated as K_h_ = K_h0_ × exp (2700 × ($\frac{1}{T} - \ \frac{1}{{298.15}}$)) (mol m^−3^ pa^−1^); K_h0_ is Henry's law constant at the standard temperature (T = 298.15), which is equal to 2.4 × 10^−4^ (mol m^−3^ pa^−1^); R is the ideal gas constant, which is equal to 8.314472 m^3^ Pa/(K mol); and T is the water temperature (K).

### Measurements and processing of N_2_O isotopic signatures

In recent years, stable isotopes have been widely used to reveal the underlying mechanism of riverine N_2_O emissions [19,20,61,62], which offer quantitative information on N transformation and N_2_O sources. To obtain the δ^15^N^Bulk^, δ^18^O and δ^15^N^SP^ values of N_2_O, the gas samples were introduced into an isotope ratio-monitoring mass spectrometer (GC-IRMS; Delta V Plus, Thermo Fisher Scientific, Bremen, Germany). Calibration was conducted by measuring the N_2_O standards (330 ppb; δ^15^N^α^, −0.4‰; δ^15^N^β^, −0.15‰; δ^15^N^Bulk^, −0.28‰; δ^18^O, 41.95‰; Air Liquide America, Specialty Gases LLC). The isotope ratios of N_2_O in delta (δ) notation were used for analysis and are presented in units of per mil (‰), as expressed in Eqs. 2–5:


(2) \begin{eqnarray*}
{{\delta }^{15}}{{{\mathrm{N}}}^{{\mathrm{Bulk}}}} &=& \Big[ {{{{\left( {^{15}{\mathrm{N}}{{/}^{14}}{\mathrm{N}}} \right)}}_{{\mathrm{sample}}}}/{{{\left( {^{15}{\mathrm{N}}{{/}^{14}}{\mathrm{N}}} \right)}}_{{\mathrm{reference}}}} - 1} \Big]\nonumber\\
&&\, \times 1000
\end{eqnarray*}

(3) \begin{eqnarray*}{{\delta }^{15}}{{{\mathrm{N}}}^\beta } = 2 \times {{\delta }^{15}}{{{\mathrm{N}}}^{{\mathrm{Bulk}}}} - {{\delta }^{15}}{{{\mathrm{N}}}^\alpha }\end{eqnarray*}

(4) \begin{eqnarray*}{{\delta }^{15}}{{{\mathrm{N}}}^{{\mathrm{SP}}}} = \ {{\delta }^{15}}{{{\mathrm{N}}}^\alpha } - {{\delta }^{15}}{{{\mathrm{N}}}^\beta }\end{eqnarray*}

(5) \begin{eqnarray*}
{{\delta }^{18}}{\mathrm{O}} &=& \left[ {{{{\left( {^{18}{\mathrm{O}}{{/}^{16}}{\mathrm{O}}} \right)}}_{{\mathrm{sample}}}}/{{{\left( {^{18}{\mathrm{O}}{{/}^{16}}{\mathrm{O}}} \right)}}_{{\mathrm{reference}}}} - 1} \right]\nonumber\\
&&\, \times 1000.
\end{eqnarray*}

However, the exchange of N_2_O between atmosphere and water alters the microbial isotopic signatures of N_2_O in the water samples (δ_i, water_). A Keeling plot, based on the conservation of mass, can be applied to obtain the microbial isotopic signatures of N_2_O and avoid bias derived from atmosphere-water exchange [63–65]. It assumes that the measured isotopic signature is a mixture of background atmosphere values and the microbial additions. The concentrations and isotopic signatures of dissolved N_2_O were plotted with the atmospheric ones (δ^15^N^Bulk^ = 6.55 ± 0.47, δ^18^O = 44.4 ± 0.34 and δ^15^N^SP^ = 19.4 ± 1.92) [49]. The isotopic signatures of microbially produced N_2_O are represented as the y-intercept value. In addition, it is worth noting that N_2_O may undergo reduction processes, which can significantly alter their original isotopic signatures (δ_i, original_). During N_2_O reduction, the ratio between the isotope effects for δ^15^N^SP^ and δ^18^O remained relatively stable. Thus, the δ^15^N^SP^/δ^18^O map could be used to quantify the degree of N_2_O reduction and calculate the original isotope source signatures (Fig. S5) [66,67].

### Bayesian isotope mixing model

N_2_O microbial sources can be quantified by distributing *in situ* isotopic signatures to each microbial process [48–50], which was realized by the Stable Isotope Mixing Models in R (SIMMR) [68]. The Stable Isotope Mixing Models were formulated as Eqs. 6–9:


(6) \begin{eqnarray*}{{X}_{ij}} = \frac{{\mathop \sum \nolimits_{k = 1}^K {{p}_k}{{q}_{jk}}({{s}_{jk}} + {{c}_{jk}})}}{{\mathop \sum \nolimits_{k = 1}^K {{p}_k}{{q}_{jk}}}} + {{\varepsilon }_{ij}}\end{eqnarray*}

(7) \begin{eqnarray*}{{s}_{jk}} \sim N ( {{{\mu }_{jk}},\omega _{jk}^2} )\end{eqnarray*}

(8) \begin{eqnarray*}{{c}_{jk}} \sim N ( {{{\lambda }_{jk}},\tau _{jk}^2} )\end{eqnarray*}

(9) \begin{eqnarray*}{{\varepsilon }_{ij}} \sim N ( {0,\sigma _j^2} ),
\end{eqnarray*}

where ${{X}_{ij}} = $ observed isotope value *j* of the mixture *i* (i.e. δ_i, original_); ${{s}_{jk}} = $ source value *k* on isotope *j*, normally distributed with mean ${{\mu }_{jk}}$ and variance $\omega _{jk}^2$ (i.e. corrected values of denitrification, NDN and nitrification in Table S3); ${{c}_{jk}} = $ fractionation factors for isotope *j* on source *k*, normally distributed with mean ${{\lambda }_{jk}}$ and variance $\tau _{jk}^2$. As the original isotope source signatures of N_2_O before reduction were input in this study, the isotope fractionation factors ${{c}_{jk}} = $ 0; ${{p}_k} = $ proportion of source *k*, estimated by the model; ${{q}_{jk}} = $ concentration of isotope *j* in source *k*; ${{\varepsilon }_{ij}}{\mathrm{\ = }}$ residual error, describing additional inter-observation variance not described by the model, while $\sigma _j^2$ was estimated by the model.

Because δ^15^N^Bulk^ and δ^18^O are dependent on the substrates, the exchange of O between H_2_O and precursors of N_2_O also perturbs δ^18^O of N_2_O, therefore we adopted corrected isotopic values to avoid bias from precursor substances [66,69]. The Kjeldahl distillation procedure was employed to measure the stable isotope ratios of δ^15^N-NH_4_^+^ [70]. NO_3_^−^ was transformed into N_2_O for determination of the δ^15^N-NO_3_^−^ and δ^18^O-NO_3_^−^ isotopes using denitrifying bacteria (*Pseudomonas aureofaciens*; ACTT 13985, USA). The isotopic signatures for each microbial process and precursors are listed in Table S3.

### Management strategies

The difference in the N_2_O partial pressure (ΔN_2_O = N_2_O_water_ – N_2_O_equilibrium_) for global rivers was used to indicate the N_2_O emission capacity. The air-water N_2_O equilibrium concentration (N_2_O_equilibrium_) can be calculated via Eq. 10 [71]:


(10) \begin{eqnarray*}{{{\mathrm{N}}}_2}{{{\mathrm{O}}}_{{\mathrm{equilibrium}}}} = {{{\mathrm{K}}}_{\mathrm{h}}} \times {{{\mathrm{p}}}_{\mathrm{A}}} = {{{\mathrm{K}}}_{\mathrm{h}}} \times {{{\mathrm{C}}}_{\mathrm{A}}}{\mathrm{RT}},\end{eqnarray*}

where N_2_O_equilibrium_ is the air-water equilibrium concentration of N_2_O (nmol/L) and C_A_ is the atmospheric N_2_O concentration. In the absence of local water temperature data, the existing saturation data of N_2_O (S = N_2_O_water_/N_2_O_equilibrium_ × 100%) were employed to calculate the ΔN_2_O, ΔN_2_O = N_2_O_water_ × (S-100%)/S.

Two scenarios were established for managing riverine N_2_O emissions. Under Scenario 1, the reduction proportion for restoring hotspots to their baselines was calculated via Eq. 11:


(11) \begin{eqnarray*}
&& \sum (\Delta {{{\mathrm{N}}}_2}{{{\mathrm{O}}}_{{\mathrm{i}},{\mathrm{hotspots}}}} - \Delta {{{\mathrm{N}}}_2}{{{\mathrm{O}}}_{{\mathrm{A}}/{\mathrm{U}}\,{\mathrm{baseline}}}})/ \nonumber\\
&&\quad \sum \Delta {{{\mathrm{N}}}_2}{{{\mathrm{O}}}_{\mathrm{i}}},
\end{eqnarray*}

where ΔN_2_O_i, hotspots_ is the ΔN_2_O concentration at each hotspot; ΔN_2_O_A/U baseline_ is the median ΔN_2_O concentration in agricultural (23.9 nM) and urban (21.7 nM) rivers; and ΔN_2_O_i_ is the ΔN_2_O concentration at each sampling point in agricultural and urban rivers.

Under Scenario 2, a nitrate-based ΔN_2_O reduction model was established, and restoration was performed from the highest unit benefits (slope, ΔN-N_2_O/N-NO_3_^−^). The EF_Control_, median concentration, unit benefits and reduction proportion were calculated for the hotspots and baseline points at 10% intervals (Table S4). For each group of sample points (e.g. 10%–20%), EF_Control_ is the lowest slope (ΔN-N_2_O/N-NO_3_^−^) at the final order. Moreover, the unit benefit is the average ΔN-N_2_O/N-NO_3_^−^ value in this group. The reduction proportion for restoring the agricultural/urban hotspots and baselines to natural baselines was calculated via Eqs. 12 and 13, respectively:


(12) \begin{eqnarray*}\sum (\Delta {{{\mathrm{N}}}_2}{{{\mathrm{O}}}_{{\mathrm{i}},{\mathrm{hotspots}}}} - \Delta {{{\mathrm{N}}}_2}{{{\mathrm{O}}}_{{\mathrm{Natural}}\,{\mathrm{baseline}}}})/\sum \Delta {{{\mathrm{N}}}_2}{{{\mathrm{O}}}_{\mathrm{i}}}\end{eqnarray*}

(13) \begin{eqnarray*}\sum (\Delta {{{\mathrm{N}}}_2}{{{\mathrm{O}}}_{{\mathrm{i}},10\% }} - \Delta {{{\mathrm{N}}}_2}{{{\mathrm{O}}}_{{\mathrm{Natural}}\,{\mathrm{baseline}}}})/\sum \Delta {{{\mathrm{N}}}_2}{{{\mathrm{O}}}_{\mathrm{i}}},
\end{eqnarray*}

where ΔN_2_O_i_,_hotspots_ is the ΔN_2_O concentration at each hotspot; ΔN_2_O_i_,_10%_ is the ΔN_2_O concentration at the sampling points in agricultural and urban rivers at 10% intervals; ΔN_2_O_Natural baseline_ is the median ΔN_2_O concentration in natural rivers (0.02 nM); and ΔN_2_O_i_ is the ΔN_2_O concentration at each sampling point in agricultural and urban rivers.

## Supplementary Material

nwae458_Supplemental_Files

## Data Availability

All data used in this study are compiled into Supplementary data. Correspondence and requests for materials should be addressed to Guodong Ji (jiguodong@pku.edu.cn).
